# A Remember/Know Examination of Free-recall Reveals Dissociative Roles of Item- and Context-Information over Time

**DOI:** 10.1038/s41598-018-31401-w

**Published:** 2018-09-10

**Authors:** Talya Sadeh, Rani Moran, Yonatan Stern, Yonatan Goshen-Gottstein

**Affiliations:** 1 The Department of Cognitive and Brain Sciences, Ben-Gurion University of the Negev, Beersheba, Israel; 2 Zlotowski Center for Neuroscience, Ben-Gurion University of the Negev, Beersheba, Israel; 3 Department of Psychology, Ben-Gurion University of the Negev, Beersheba, Israel; 4 Max Planck UCL Centre for Computational Psychiatry and Ageing Research, University College London, London, UK; 5 Wellcome Centre for Human Neuroimaging, University College London, London, UK; 6 School of Psychological Sciences, Tel-Aviv University, Tel Aviv, Israel

**Keywords:** Forgetting, Long-term memory

## Abstract

It is well-established that the ability to freely recall information is driven by the extent to which the context at encoding is reinstated at retrieval. Still, when asked to judge the subjective quality of one’s memories giving Remember/Know (R/K) judgments, people tend to classify a substantial proportion of recalls as being devoid of context. We suggest that R- and K-recalls differ with regard to their reliance on context- and item-information, with R-recalls driven primarily by contextual-information (e.g., associations evoked by the study-items) and K-recalls driven primarily by information pertaining to the items (e.g., semantic information). Memory was tested both immediately after study and in a final free-recall test conducted ~20 minutes after encoding—a timescale which is akin to real-life events. In line with our predictions, as compared to K-recalls, R-recalls show stronger contextual effects, but similarly strong item-related effects over these timescales. Furthermore, drawing on theories regarding the forgetting of item- and contextual information, we hypothesized and found that R- and K-recalls are differentially affected by the passage of time. Our findings provide several converging pieces of evidence for differential roles of item and contextual information in driving recall and thus highlight the need to extend longstanding theories of free-recall to account for cases in which recall relies less on context.

## Introduction

Ever since Ebbinghaus’ first laboratory investigations, free-recall has been a paramount experimental paradigm in unraveling mechanisms of episodic memory^1–4^. In free-recall, participants are presented with a list of items (typically words) and are subsequently required to retrieve them in any order. Successful recall requires an active, self-initiated, search process for the items, with no external retrieval cues. Most theoretical models of free-recall focus on the role of context in driving recall^5–10^. Such context typically refers to an amalgam of the external environment, an individual’s stream of thoughts and associations evoked by the study-items^6^.

While the importance of context in recall cannot be overstated, a number of studies have shown that a substantial proportion of recalled items (between 12–42%^11–17^) are judged by participants as being devoid of contextual associations. These findings were obtained by combining the Remember/Know (R/K) paradigm with free-recall (for similar results in paradigms using the process dissociation procedure see^18,19^). In the R/K paradigm, participants are required to give a subjective judgment regarding each item they retrieve: a “Remember” (R) judgment to indicate that retrieval of the item was accompanied by contextual associations (namely, information in addition to the item itself was recalled) and a “Know” (K) judgment to indicate knowledge that the retrieved item was presented at study, even though its retrieval was not accompanied by any contextual associations. Notably, a substantial proportion of items recalled are given a K-judgment even when participants are instructed to make this judgment only if they are absolutely certain that they cannot retrieve any contextual details and that it is acceptable to give no K-responses at all^17^. The current study is aimed at furthering our understanding of the retrieval process underlying recalls given a K-response—K-recalls—and the manner in which it differs from the retrieval process underlying R-recalls.

Recently, it has been suggested that as compared to K-recalls, R-recalls rely more on contextual-information—namely source information regarding the item’s environment or the associations it evoked^14,17^. For example, memory for source information—which of two encoding tasks was performed with each item—was found to be more accurate for R-recalls than for K-recalls^14^. Furthermore, in a recent study conducted in our lab^17^, we found R-recalls to exhibit a larger Temporal-context-effect than K-recalls. The Temporal-context-effect^3,20,21^ (a.k.a The Temporal-Contiguity Effect) refers to the clustering together at retrieval of items which had neighboured at encoding. This effect is believed to occur due to the largely overlapping contexts of items that were studied in close temporal proximity, as compared to items that were studied further away from each other. It has been argued that the temporal context—thoughts and associations evoked by the study-item—that is retrieved along with the recall of an item triggers the recall of neighbouring items, which had been bound to a similar context. Our findings thus suggested that as compared to R-recalls, K-recalls rely less on context reinstatement. Instead, K-recalls may reflect retrieval triggered predominantly by item-information^14,17^ (see^22^ for fMRI evidence for the role of item-information in driving recall). That is, K-recalls are driven by information related to the item itself—e.g., information regarding semantic attributes of the item—rather than the context in which it was presented. Phenomenologically, K-recalls are experienced as an item ‘popping into mind’^17^ with no accompanying context.

An alternative to the above item/context interpretation of R- and K-recalls is that—rather than showing qualitative differences in the type of information cuing recall—the two responses differ quantitatively in their underlying memory strengths. According to this strength interpretation, K-recalls merely reflect cases in which the memory traces of the recalled items are weaker, as compared to those of R-recalls. Such interpretations have often been raised as an account for differences between R and K responses in recognition paradigms^23–25^ (but see^26^).

The primary goal of the current study was to differentiate between the item/context account and the strength account of R- and K-recalls. We aimed to extend previous evidence that R- and K-recalls differ with regard to their reliance on item- and contextual-information in two important ways. First, the few previous studies which compared R- and K-recalls focused only on contextual effects (e.g., source memory^14^ and Temporal-context-effect^17^). In contrast, in the current study we compared R- and K-recalls with regard not only to contextual effects, but to item-related effects as well. To this end, we used two measures of recall dynamics which are indicative of two types of information—item and contextual—that drive recall.

Second, we aimed to bolster the ecological validity of such evidence by examining memory for events unfolding over longer timescales, which are more akin to real-life events. Indeed, while previous studies investigating contextual and non-contextual processes in recall examined mnemonic effects which spanned several seconds, real-life events usually unfold over longer timescales, comprising at a minimum several minutes^27–29^. To examine R- and K-recalls over longer time, participants studied and were tested on 25 lists of words using free-recall (henceforth, short-delay tests). This was followed, at the very end of the experiment, by a surprise final-free-recall test^30–33^ in which participants were asked to recall items from any of the 25 lists in any order. In both short-delay tests and in the final free-recall test, retrieval of each word was followed by an R/K judgment. The data on the short-delay free-recall tests were described in Sadeh *et al*.^17^. Here we only focus on the final free-recall test and its comparison to the short-delay tests.

Our first prediction regards recall dynamics indicative of contextual information, probed via the Temporal-context-effect. To reiterate, this effect pertains to the finding that items which neighboured at encoding are more likely to cluster together at retrieval. In contrast to our previous study which examined the Temporal-context-effect for items presented within the same list^17^, here we explored the recall dynamics of items across lists—the probability of making transitions to neighboring lists, which were presented several minutes apart, as well as transitions within the same list. We predicted a stronger Temporal-context-effect in Final Free-recall for R-retrievals than for K-retrievals. This prediction is derived directly from the item/context account and is difficult to reconcile with a strength account. Thus, the Temporal-context-effect is accounted for in terms of reliance on contextual processing, rather than reflecting stronger memory traces^34^.

To examine the effects of item-information, we leveraged the fact that the encoding task used in the current study required participants to answer a question related to semantic properties of the item itself (does the studied-word represent an abstract or concrete concept?). We could thus examine the clustering, at recall, of words that were given the same response (abstract or concrete). We expected that overall—across R and K responses—recall of a word which was given an ‘abstract’ response would more likely trigger subsequent recall of an additional word given an ‘abstract’ response, as compared to a word given a ‘concrete’ response (and vice-versa). We henceforth refer to this predicted effect as “Item-related-clustering-effect”.

Critically, we predicted that the Item-related-clustering-effect would show a different pattern as compared to the Temporal-context-effect—a contextual-related clustering effect. Because K-recalls rely on item-information to the same extent as (or even more than) R-recalls, they should show a similar magnitude of Item-related-clustering as R-recalls, or even a larger effect. As we further elaborate in the Discussion, the predicted patterns of clustering are derived directly from the item/context account of R- and K-recalls and are not compatible with a strength account. Thus, the item/context account predicts differential patterns of clustering for R- and K-recalls: greater temporal clustering for R than for K, but greater (or similar in magnitude) Item-related-clustering-effects for K than for R. In contrast, a strength account would predict greater effects for R-recalls than for K-recalls in all clustering measures.

Additional support for the item/context account could be found by examining changes in the two types of recall over time. The crux of this idea is that changes in memories over time depend on the relative contribution of item- and contextual-information to their representations^35–38^. In particular, contextual-information is short-lived and is more rapidly forgotten than item-information^35,36,39–41^. If R- and K-recalls differ with regard to their reliance on item and contextual information, as the item/context account maintains, then R- and K-recalls should be differentially affected by the passage of time. Below we detail two specific predictions that ensue from this notion.

The first of these predictions regards forgetting over time. The current design enabled us to examine a direct manifestation of forgetting: the list-recency effect. This effect pertains to the better memory, in final free-recall, for items that had been presented in recently-studied lists as compared to items that had been presented in earlier lists^31,32^. The list-recency effect is thought to occur due to the decay of an item’s context with the increase in temporal distance between its encoding and retrieval^42^. Here we suggest a novel possible exception to this phenomenon—that *only* memories bound to context should be forgotten as a function of time. In contrast, non-contextual memories, which we conceptualize as relying primarily on item-information, would be less (or differentially) affected by time. We, therefore, predicted that R-recalls, by virtue of relying predominantly on context, would show a robust list-recency effect. K-recalls, in contrast, were predicted to show a reduced list-recency effect or no effect at all. This predicted pattern is not easily reconciled with a strength account. That is, why would stronger memory traces be more sensitive to forgetting over time than weaker memory traces?

Our final prediction regarding the effects of time on R- and K-recalls pertains to different patterns of reminiscence^43^. Reminiscence refers to the successful retrieval of items following failure to retrieve them in a previous attempt. In the current instance, reminiscence refers to the recall of items in the final free-recall test that were initially forgotten in the short-delay test—henceforth referred to as *novel-recalls*. Novel-recalls show a counter-intuitive relationship with time. These items are forgotten after a short delay but after a long delay, when the context should have decayed, are remembered. We suggest that novel-recalls reflect items for which the context had not been well encoded at study. Failure to retrieve these items was, therefore, a consequence of the inability to utilize their encoding context as a retrieval cue. If so, their later retrieval during the final free-recall test may have been mediated by a cue other than that of context. Thus, we predicted that the proportion of novel-recalls out of all retrievals will be larger for K- than for R- recalls.

## Results

### General

In the final free-recall test, participants correctly recalled a mean of 63.74 (SEM = 3.22; 21.25%) words. Of the words recalled, a mean of 70.85% were judged as R and a mean of 29.15% as K. Of the words recalled in the final free-recall test, 28% (SEM = 1.4%) were given an “abstract” response at encoding and 65% (SEM = 1.7%) were given a “concrete” response. The remaining items were not given any response.

We now describe the results corresponding to our different predictions. For some of these analyses, there were several participants with insufficient data points for either R or for K. These participants were excluded from these analyses, as is reflected in their lower degrees of freedom.

#### Prediction 1. Recall dynamics of R- and K-recalls: Temporal-context-effect

The Temporal-context-effect was indexed by Temporal-clustering scores introduced by Polyn *et al*.^6^ (for full details see Methods). Temporal-clustering scores are obtained by ranking each participant’s across-list recall transitions according to the extent to which they follow the temporal positions of items during study. These scores range between 0 and 1. A score of 0.5 indicated that only half of the times a participant made ‘temporal-context-utilized’ transitions, thus indicating chance-level organization. A score of 1 indicates that all of the transitions were optimal in terms of the temporal context.

**Figure 1:**
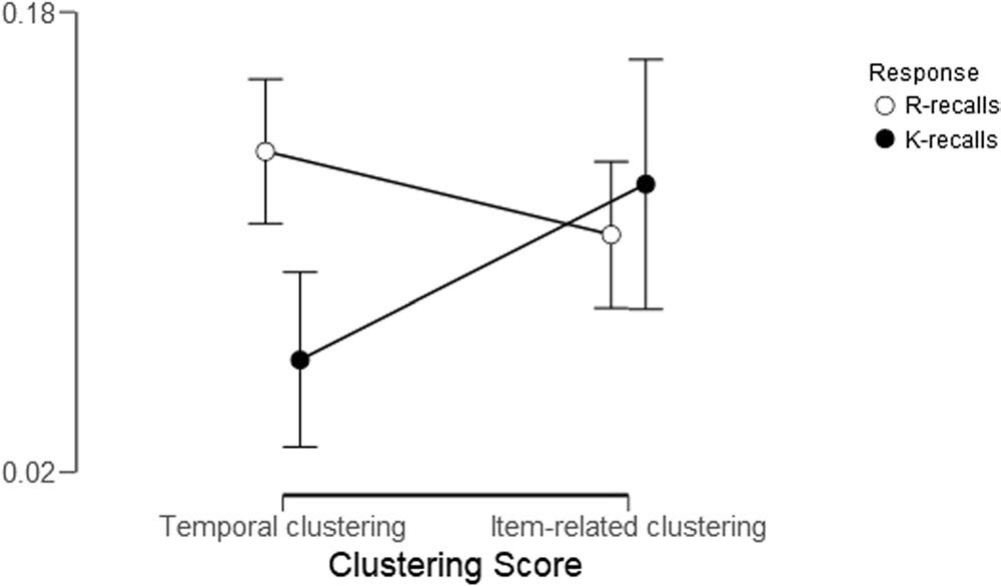
Recall dynamics’ scores for R- and K-recalls. Temporal-clustering scores denote the tendency to successively recall two items which were temporally-adjacent at study. Item-related-clustering scores measure the tendency of successively recalling two items which were given the same judgment at encoding (either ‘abstract’ or ‘concrete’). The measure of clustering scores is the difference between the raw clustering scores and the baseline scores, with a score of 0 representing chance. Thus, all clustering scores are significantly above chance level. Error bars represent 95% confidence intervals around the mean.

The across-participant mean Temporal-clustering score for R-recalls was 0.63 and for K-recalls was 0.56. For both R- and K-recalls the Temporal-clustering scores were significantly above chance level, as revealed by a one-sample t-test against 0.5 (which indicates chance). For R: t_76_ = 12.26, p < 0.001, η²_p_ = 0.66; For K: t_76_ = 4.27, p < 0.001, η²_p_ = 0.19). The mean difference between the Temporal-clustering scores and the baseline scores for R and K are presented in Fig. 1. Importantly, in line with our prediction, the mean Temporal-clustering score for R-recalls was significantly larger than for K-recalls (t_76_ = 4.6, p < 0.001, η²_p_ = 0.22). Out of 77 participants, 56 showed an effect in the ‘R > K’ direction. This result was found to be statistically-significant in a binomial test (p < 0.0001).

An additional analysis of Temporal-context-effects (see Supplemental Information) was conducted to control for confounding factors of list-recency (as well as fluctuations in attention), as described by Howard *et al*.^30^. This analysis confirmed that the obtained Temporal-context-effects could not be accounted for by these confounding factors.

A possible concern is that the results may be mediated by the larger proportion of R-recalls as compared to K-recalls. To examine this possibility, we performed a non-parametric permutation test, using a random-relabeling process, 5,000 times per participant. For each participant, the relabeling process maintained the number of observed R and K items in the body of the final free recall data. For each of the 5,000 relabeling iterations, we calculated the Temporal-clustering score for relabeled-R and relabeled-K-recalls. The across-participant mean difference between the relabeled-R and relabeled-K Temporal-clustering scores was calculated. This resulted in a distribution of 5,000 across-participant mean differences—the mean of which was 1.58E-05. None of the 5,000 across-participant means was larger than the across-participant mean calculated on the original data (0.072). Thus, it seems difficult to defend the notion that the obtained difference in temporal-clustering scores between R- and K-recalls was mediated by the higher proportion of R recalls in our data.

#### Prediction 2. Item-related-clustering-effects

Item-related-clustering scores reflect the tendency of successively recalling two items which were given the same judgment at encoding (either ‘abstract’ or ‘concrete’). For both R- and K-recalls, the clustering score were significantly above baseline (i.e., chance—no effect of clustering; Fig. 1). For Item-related clustering, an individual baseline score is calculated per participant (see Methods for details). For R-recalls, the across-subject mean Item-related-clustering score was 0.62, while the baseline score was 0.52 (t_71_ = 7.8, p < 0.001, η²_p_ = 0.46). For K-recalls, the across-subject mean Item-related-clustering score was 0.64, while the baseline score was 0.52 (t_70_ = 4.98, p < 0.001, η²_p_ = 0.26). As illustrated in Fig. 1, on a descriptive level, K-recalls showed stronger effects of Item-related clustering than R-recalls.

To test whether the difference between R- and K-recalls is significant, we computed, for each participant, the difference between her Item-related-clustering score and the baseline score. The mean difference score for R-recalls (=0.1) was not significantly smaller than that for K-recalls (=0.12; t_70_ = 0.7, p = 0.48, η²_p_ = 0.007). This was further confirmed by a Bayesian paired-sample t-test which revealed moderate evidence in favour of the null hypothesis (B_01_ = 6.04).

Finally, we confirmed our prediction that Item-related-clustering-effects would show a different modulation by response type (R, K) as compared to the Temporal-context-effect. As illustrated in Fig. 1, while R-recalls showed a larger Temporal-context-effect than K-recalls—namely, a larger contextual-related clustering effect—this was not the case for the Item-related-clustering-effect. To test the statistical significance of this pattern, we ran a repeated-measures ANOVA with clustering type (Temporal-context-effect, Item-related-clustering) and response type (R, K) as within-participant factors. The dependent variable was the difference score between the clustering scores and the baseline scores. A significant interaction was found between clustering and response type (F_1,70_ = 9.1, MSE = 0.016, p = 0.004, η²_p_ = 0.12).

#### Prediction 3. The List-Recency Effect

**Figure 2:**
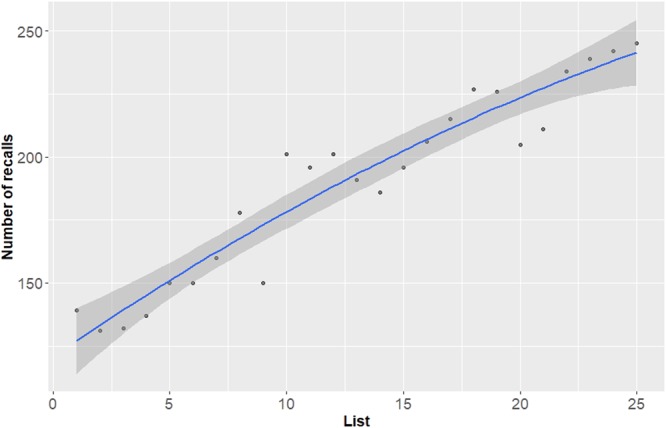
The List-Recency effect. Total number (across all participants) of words recalled per list, collapsed across R and K. A classic list-recency is demonstrated, with higher probability of recalling items from the final lists as compared to the first and middle lists. The blue line denotes the regression fit.

To establish that our results replicated the classic list-recency effect^30–33^, we examined the number of items recalled for each of the lists—collapsed across R and K responses. We found a stronger tendency for recalling words from more recent lists as compared to words from more distant lists (Fig. 2).

**Figure 3:**
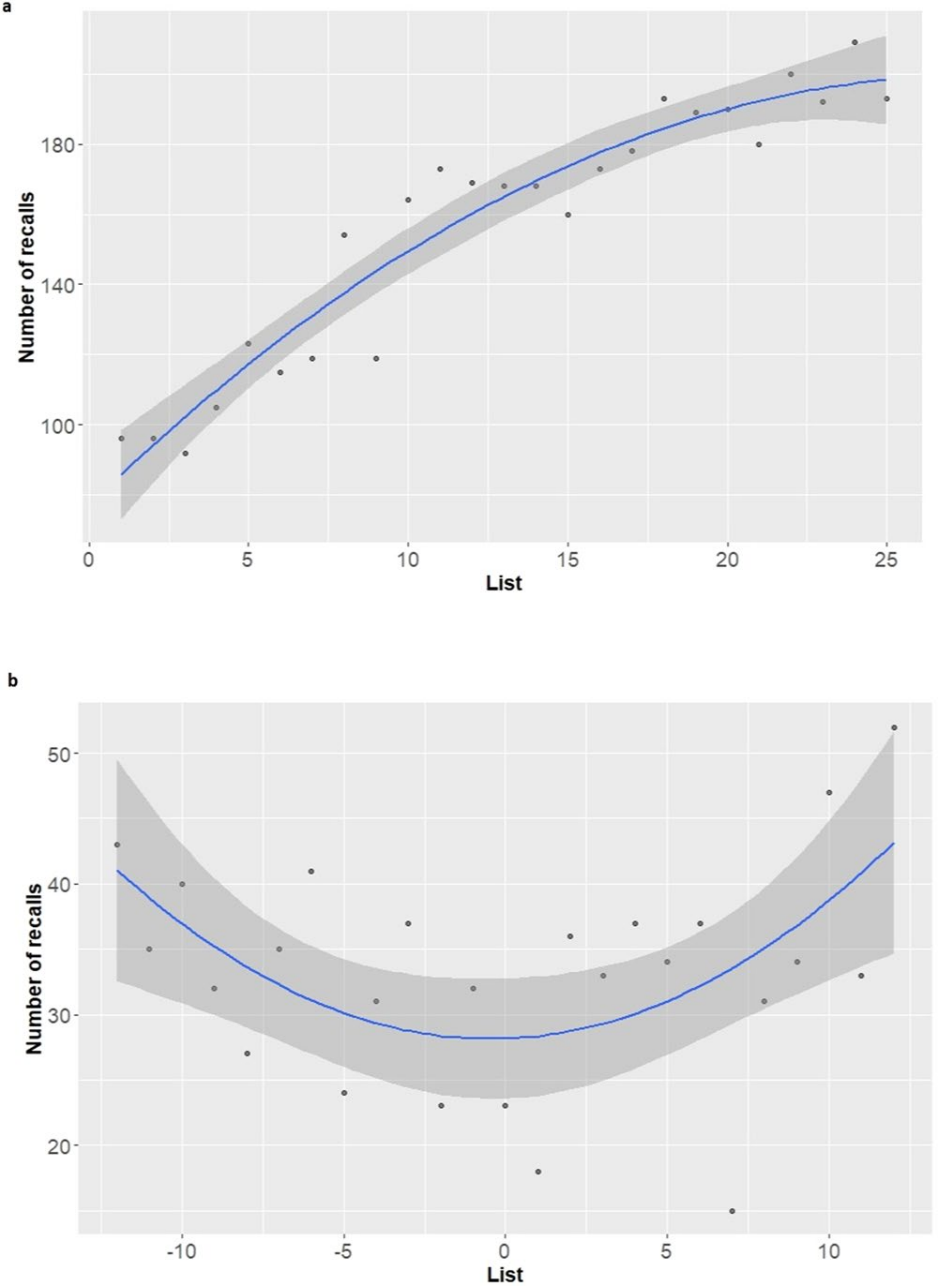
The list-recency effect for R and K. Total number (across all participants) of R-recalls (**a**) and K-recalls (**b**). R-recalls exhibit a classic list-recency, with higher probability of recalling items from the final lists as compared to the first and middle lists. K-recalls exhibit a U-shaped function: higher probabilities of recalling items from both first and final lists, as compared to middle lists. Blue lines denote the regression fits.

Next, we compared the list-recency effects for items given R and K responses during the short-delay tests. This analysis thus asked whether items that were initially judged as R were more likely to show a decrease in recall over time (namely, with distance from study) as compared to items judged as K. Figure 3 presents the distributions of R- and K-recalls across lists. For each list, the total number of recalls was summed across all participants, separately for R- and K-recalls. The figure reveals that K-recalls do not show the same monotonic increase in recall probability as a function of list-number, confirming Prediction 1.

Visual inspection of the curves of total recall as a function of list number suggests that they may be non-linear. We, therefore, regressed total recall per list on list number. Our regression model included both linear and quadratic terms for list number, response (R or K) and the interactions between the (linear and quadratic) list number terms and response. We found that the quadratic list-number term interacted with recall type (beta = −0.256, t_44_ = −4.48, p < 0.001), indicating that total R- and K-recalls differed in their quadratic relation to list number. We, therefore, proceeded to examine the simple list number effects for R and for K. We found a negative list-number quadratic effect for R (beta = −0.159, t_44_ = −3.936, p < 0.001), indicating that total R-recall increased, albeit with a decreasing rate, with list number (see Fig. 3 for the regression fit). Unlike R, we found a positive list-number quadratic effect for K (beta = 0.097, t_44_ = 2.4, p = 0.021), indicating that the total number of K-recalls showed a nonmonotonic U-shape curve as a function of list-number (see Fig. 3 for the regression fit).

These findings reveal that K-recalls did not show the same monotonic decrease in recall probability with distance from study, as did R-recalls. Thus, in line with our a-priori prediction, the classical list-recency effect was observed for R-recalls, but not for K-recalls.

#### Prediction 4. Novel-recalls

The proportion of novel-recalls (collapsed across R and K) out of the total number of recalls in the final free-recall test was 20.7% (SEM = 0.13%). In line with our prediction, we found that whereas 39.7% of K-recalls were novel (out of all K-recalls in the final free-recall test), only 14.3% of R-recalls (out of all R-recalls) were novel. The difference between the proportions of R and K was significant (t_77_ = 10.64, p < 0.001, η²_p_ = 0.595).

Importantly, if K-recalls are output later than R-novel recalls, this result might be compatible with the strength account, as weaker items are believed to be retrieved later in the recall sequence^44,45^. Indeed, paired-sample t-tests revealed that R-items were output earlier than K-items (Mean R output = 35.42; Mean K output = 47.47; t_77_ = −7.23, p < 0.001, η²_p_ = 0.404).

We, therefore, examined whether the difference in proportions of novel-recalls between R- and K-recalls was evident even after accounting for differences in output positions between the two response types. To do so, for each participant we calculated the difference between the mean output positions of K-recalls and the mean output positions of R-recalls. The difference in mean output positions was included as a covariate in a repeated-measures ANOVA with the proportions of novel-recalls as the dependent variable and response type (R, K) as a within-participant factor. The interaction between response type and difference in mean output position was significant (F_1,75_ = 8.79, MSE = 0.021, p = 0.004, η²_p_ = 0.105), indicating that the effect of response type on novel-recalls proportions was partially mediated by differences in output positions between R- and K-recalls. Most importantly, however, the main effect of response type remained significant (F_1,75_ = 43.43, MSE = 0.021, p < 0.001, η²_p_ = 0.367). Thus, the difference in proportions of novel-recalls between R- and K-recalls cannot be fully accounted for by differences in output positions between the two response types. Rather, it seems necessary to advocate for the item/context account.

An additional analysis was conducted to further support the notion that novel-recalls do not merely reflect weaker memory traces than non-novel recalls, but rather that the two conditions differ with regard to their reliance on item- and context-processing. To this end, we examined both temporal clustering and Item-related-clustering effects of non-novel compared to novel recalls. As in our previous analysis of clustering scores, all scores reported are difference scores, that is, we subtracted the baseline score from the raw clustering scores.

We found that temporal clustering scores were significantly smaller for novel-recalls as compared to non-novel recalls (Mean difference score of novel-recalls = 0.052; Mean difference score of non-novel recalls = 0.136; t_87_ = −5.47, p < 0.001, η²_p_ = 0.26). This result is in line with the notion that novel-recalls reflect items for which the context had not been well encoded at study and therefore their retrieval during the final free-recall test was mediated by a cue other than that of context.

In contrast to temporal clustering scores, no difference was found in Item-related clustering scores (Mean difference score of novel-recalls = 0.083; Mean difference score of non-novel recalls = 0.105; t_87_ = −0.81, p > 0.4, η²_p_ = 0.008). This null result was confirmed by a Bayesian paired-sample t-test which revealed that given our data, the null hypothesis was 6.18 times more likely than the alternate hypothesis Finally, a significant interaction was found between clustering (Temporal, Item-related) and novelty (novel, non-novel; F_1,87_ = 4.09, MSE = 0.021, p = 0.046, η²_p_ = 0.045). These results thus further establish that novel and non-novel recalls differ in their reliance on item and context information. Namely, that the item/context account captures the distinction between these types of recall better than the strength account.

#### Auxiliary Analysis. A comparison between short and long delays

An auxiliary analysis we conducted was aimed at testing the following notion: assuming that context decays over time and is more rapidly forgotten than item information, the proportion of R-recalls from all recalls (i.e., from the sum of R- and K-recalls) would be higher when there is a short delay between study and test, as compared to a longer delay. We thus hypothesized that the proportion of R-recalls in the short-delay tests—the free-recall tests following the presentation of each of the lists along with its 30 s arithmetic distractor task—should be higher than the proportion of R-recalls in the Final Free-recall test. Note that in contrast to the other predicted patterns, this pattern is also compatible with a strength account.

In line with our prediction, a larger proportion of recalls were given an R response in the short-delay free-recall tests (76%; SEM = 2.23%) than in the final free-recall test (71%; SEM = 2.77%). The difference between these proportions was significant (t_87_ = 2.82, p = 0.006, η²_p_ = 0.084).

## Discussion

The current study provides multiple lines of evidence in support of the notion that R- and K-recalls qualitatively differ with regard to their reliance on context- and item-information. Most importantly, we found differential dynamics of retrieval for R- and K-recalls, which are indicative of differential reliance on contextual and item information. Two effects were found in this regard: (1) The Temporal-context-effect, which refers to context-related clustering effects and measures the clustering together, at recall, of items that were studied in close temporal proximity and (2) The Item-related-clustering-effect, indexed by the likelihood of participants to successively recall two items which were given the same semantic judgement (abstract or concrete) at study. As predicted, these two clustering effects were differentially modulated by response type (R, K): whereas R-recalls showed a greater Temporal-context-effect than K-recalls, this was not the case for the Item-related-clustering-effect which did not differ between the two response types. Our results confirm that R-recalls rely on contextual reinstatement to a greater extent than K-recalls, but that both types of recalls rely on item-information to the same degree.

Our measure of Item-related-clustering is reminiscent of—but not identical to—‘source clustering’^6^. The measure of source clustering is obtained when source-information is manipulated at study by having participants encode each item in one of two encoding tasks (e.g., size/pleasantness judgment). Source clustering documents the effect that participants tend to successively recall two items that were encoded with the same task—for instance, two items that were encoded with a pleasantness judgment. Critically, the similarity between source- and Item-related-clustering is only superficial. In source clustering, the similarity between two successively-recalled items regards information external, or peripheral to the item (the encoding-task)—‘source-related features’^6^. In contrast, in Item-related-clustering, the similarity pertains to semantic information regarding the items themselves (i.e., whether they are abstract or concrete entities). Our a-priori hypothesis regarding Item-related-clustering-effects was that R- and K-recalls would not differ on this measure, or alternatively that K-recalls would show a stronger effect, because they are driven primarily by item-information. Our results support the former possibility, with evidence for the null hypothesis—namely, no difference between R- and K-recalls. This indicates that item-information plays a similarly important role in driving both R- and K-recall. In other words, while R-recalls can and typically are, driven by contextual-information, this does not attenuate the role of item-information in driving their retrieval as well. This pattern of results is directly derived from the item/context theory for R- and K-recalls. Importantly, this pattern cannot be easily reconciled with a strength account. Thus, if stronger memory traces underlay R-recalls, then item-related clustering should have been stronger for R-recalls than for K-recalls.

A strength interpretation is also not applicable to the effects of Temporal-context. Namely, it is hard to envision why a stronger tendency to recall items from nearby study positions would be found for strong—but not for weak—memory traces, without resorting to the concept of “context”. Why would retrieval of a strong memory trace be predictive of retrieval of an adjacent strong memory trace, if not through mediation of similar contexts which are associated with both items? Presumably, an item’s strength would be largely dictated by the history of an item, comprising factors such as the frequency in which it is presented, its imageability and the number of its semantic associates. However, none of these factors which affect an item’s strength, seems to be relevant to its magnitude of association with adjacent items with whom the only relationship is their shared, one-shot, episodic, contextual history.

Because the notion of strength is devoid of an item’s episodic contextual history, it does not seem reasonable to use a strength mechanism to account for our differential temporal-context effects, which epitomize contextual processing. Indeed, that a contextual interpretation seems more plausible is also reflected by the interpretation given by Schwartz *et al*.^46^ to their findings of different magnitudes of Temporal-context-effect in recognition memory as a function of confidence ratings. Finally, differences between R- and K-recalls were maintained with the permutation analysis we performed, which controls for differences in memory levels, namely, strength, between the two response types.

The current study also provides compelling evidence for differential patterns of forgetting and reminiscence for R- and K-recalls. Specifically, we found that R-recalls, which index retrieval linked to context, are subject to the perils of time. In contrast, K-recalls, which are dependent on context to a lesser extent, are not adversely affected by the passage of time. Specifically, while R-recalls show a robust list-recency effect—the likelihood of recall correlates negatively with distance from study, K-responses show a different pattern of recall as function of time.

Curiously, the pattern of K-recalls as a function of serial position fitted a quadratic function with pronounced primacy and recency effects. While this result might be of theoretical importance, it was not predicted a priori and therefore we can only offer some speculative insights in this regard. A possible account for both primacy and recency effects is provided by the dimensional distinctiveness model^47^. According to this model, the first and the most recent items studied are more distinct on a temporal dimension, as compared to middle items. This temporal distinctiveness gives rise to a higher probability of recall for those items. By this view, recall of K-items is driven by their enhanced distinctiveness—their “standing out” from other study-items. That these items are subjectively judged as being devoid of any contextual associations would suggest that distinctiveness of items is not directly amenable to consciousness.

An additional possible account for the primacy effect is enhanced attention to items in the first lists, compared to the middle and recent lists^48^. Interestingly, by this account, K-recalls might, in fact, be associated with stronger memory traces than R-recalls, which do not exhibit a primacy effect. Regardless of which, if any, of these theoretical accounts is more accurate, these patterns clearly do not lend themselves easily to a strength interpretation. Further research is needed to elucidate these exploratory findings, if replicated in future studies.

Our finding that R-recalls exhibit a robust list-recency effect were interpreted here in terms of forgetting. In particular, items from early lists, which had been presented a relatively long time before the final free-recall test, were more likely to be forgotten as compared the items from more recent lists. We have argued that this finding supports the notion that R-memories are forgotten due decay over time. However, decay is only one of two major factors contributing to forgetting, with interference from similar information also playing a major role^35,36^. Admittedly, the current memory task involved a high degree of interference because multiple word-lists were encoded and retrieved within a single session. However, while interference is undoubtedly a major cause of forgetting^35,36,49–51^, it is unlikely to account for the finding of an increase in R-recall probability as a function of list number. While retroactive interference (words from later lists interfering with words from earlier lists) alone would entail a list-recency pattern, proactive interference (words from earlier lists interfering with words from later lists) would entail the opposite pattern: greater forgetting of more recent words than of earlier words. In word-list paradigms as the current one, proactive interference has been shown to play a more dominant role than retroactive interference^51^. Thus, if interference were the major cause of forgetting of R-memories, a pattern opposite to that observed here would have been expected. Namely, one would expect the proportion of R-recalls to be highest for the first lists and lowest for the final lists. Forgetting of R-memories as demonstrated by the list-recency effect is, therefore, more likely to be interpreted in terms of decay. This finding provides compelling evidence for recent theories according to which forgetting of recollection-based memories is more likely to be caused by decay than by interference^35,36^. Note, that according to these theories, K-recalls are more likely to be forgotten due interference. This prediction, however, cannot be confirmed with the current data, as the study was not designed to systematically examine the effects of interference.

In cases in which reminiscence, rather than decay, is observed, stronger effects were found for K-recalls than for R-recalls, as indicated by the results of novel-recalls. Thus, K-recalls are more likely than R-recalls to show a counter-intuitive relationship with time, being forgotten after a short delay but remembered after a long delay. Importantly, we confirmed that the higher proportion of novel-recalls among K responses cannot be accounted for merely be the fact that K-recalls are output later than R-recalls. Thus, a strength account—according to which weaker items are recalled later in the sequence than stronger items—is not sufficient to explain this pattern of results. An additional analysis comparing clustering scores of novel vs. non-novel recalls further confirmed that novel-recalls do not reflect weaker memory traces than non-novel recalls. Rather, the two recall types differ with regard to their reliance on item- and context processing, as the item/context account predicts.

### Underlying mechanism of K recalls

What is the nature of the item-related information that drives recall? One possibility is that such recalls reflect a conglomerate of various types of information, all related to features of the item itself, rather than to its context. A strong candidate to play an important role is semantic information: namely, recall is driven by activation in the relevant semantic network. This idea is supported by our finding of item-related clustering as a driving force of K-recalls. Thus, K-recalls were clustered according to a dimension related to the semantic properties of the words—whether they represent an abstract or a concrete entity.

Additional support for the effects of semantic information in driving recall may be obtained in future studies which will systematically manipulate semantic-relatedness between study words. Still, in an exploratory analysis, we aimed to further explore whether semantic information drove recall by capitalizing on semantic-clustering effects. These effects pertain to the finding that successively recalled items are relatively more likely to be semantically related to one another, than semantically distant from one another^6^. Semantic Clustering Scores were computed similarly to the Temporal-clustering scores (see Methods). Unfortunately, neither R-recalls nor K-recalls showed semantic clustering effects (Mean R-score = 0.499, Mean K-score = 0.5). Failure to find a semantic clustering effect was likely due to the fact that this effect is difficult to obtain when semantic-relatedness between words in each list is not systematically manipulated^52^. It remains to be examined, therefore, whether, semantic-clustering effects show a similar pattern to those of Item-related-clustering-effects.

Similar ideas regarding the role of semantic information in recall have been raised by proponents of generate-recognize theories of recall e.g.^19^. For instance, according to the Fuzzy Trace Theory, recall may be driven at times by recovering of the semantic gist of study-items which is used to generate a set of possible candidates from the recovered semantic category^53^. A slave operation then follows, during which one of the possible candidates is selected based on familiarity-like judgments (but see^14^).

Though significantly smaller in magnitude as compared to R-recalls, K-recalls still exhibited a significant Temporal-context-effect. Thus, some residual contextual effects might also drive K-recall. However, the context that mediates performance is one which is not available to explicit recollection at the time of test and, therefore, the items retrieved via this context are subjectively judged as being devoid of context. In other words, recall driven by implicit reinstatement of context may be one of the processes contributing to K-recall. Still, this contribution is substantially weaker than the explicit reinstatement of context driving R-recalls.

To conclude, our findings reveal systematic differences between the effects of item- and context-information in driving free-recall. These differences—pertaining both to the dynamics of retrieval and to forgetting—are well captured by the R/K paradigm. Thus, the type of information that guides recall is at least partially available to awareness. Because of the predominant focus on context in free-recall research, very few studies to-date have examined free-recall in combination with the R/K paradigm, with the vast majority of R/K studies testing memory with a recognition test^35,54^. The current results thus highlight the need to extend longstanding theoretical frameworks of free-recall to more systematically account for cases in which recall relies less on context.

## Methods

### Participants

A total of 88 native Hebrew speakers (61 women) aged 19–31 years (mean 24.4) participated in the experiment. Participants gave their informed consent prior to the experiment and were compensated for their time monetarily or with course credit. All experimental procedures were approved by the Tel-Aviv University ethics committee. The study was carried out in accordance with the relevant guidelines and regulations. All participants provided written consent of willingness to participate in the study.

### Materials

The stimuli consisted of 348 Hebrew nouns 3–6 letter long (mean word length = 4.1 letters). For each participant, words were randomly divided into 29 lists of 12 words each. Of the 29 lists, four served as practice lists and 25 as test lists.

### Procedure

The experiment consisted of two stages, a short-delay free-recall stage and a final free-recall stage. In the short-delay stage, 25 study-lists were presented, each followed by a delayed free-recall test in which only words from the most recent list were to be retrieved. In the second, a surprise final free-recall test was given in which participants were required to recall as many words as possible from all study-lists.

In each of the study-lists, 12 words were presented. Words were presented on the screen, one at a time, for 1350 milliseconds followed by an asterisk for 400 ms. For each of the presented words, participants indicated by pressing the left or right keyboard button whether the word was abstract or concrete. To eliminate the effects of short-term memory^10^, following each list, an arithmetic distractor task was given for 30 seconds. Participants were informed that their verbal responses were recorded for analysis of their performance in the task. Following the arithmetic task, five question marks appeared on the screen for 90 seconds, signaling to participants to type out as many words as they could recall from the most recent list. For each word recalled, the Hebrew translation to the words “Remember” and “Know” appeared at the center of the screen, signaling participants to make an R/K judgment regarding the last word they had typed. Prior to the experiment, participants were given standard R/K instructions^55^, which were adapted for free-recall.

Upon completion of the first stage of the experiment, participants were informed of the surprise recall test and were reminded of the R/K instructions. In particular, it was emphasized that an R judgment should be given only if a contextual detail from the study episode was reinstated. Thus, if the only contextual detail participants remembered was from the free-recall tests that followed the presentation of each of the lists, they were to give a K judgment. The final free recall test lasted for as long as participants could recall words—never exceeding 20 minutes. As in the first stage, five question marks appeared on the screen and participants typed the words they recalled using the computer keyboard and made an R/K judgment regarding each word they typed.

To enhance performance, participants were told that they would be awarded monetary prizes (comparable to $200) if they reached the best scores in the experiment. Participants were given detailed instructions regarding the scoring method, which awarded points for correct responses and penalized for incorrect responses. Scoring was given both for the memory test and for the arithmetic tasks.

### Data Analysis

Analyses were performed using in-house R scripts^56^, MATLAB scripts and JASP^57^. All data and scripts can be viewed at http://bgu.ac.il/~tsadeh/LabDataScripts/FR_RK_for_paper_minimal/ReadMe_FR_RK_minimal.htm.

### Analysis of recall dynamics

#### Temporal-context-effect

Our measure of the Temporal-context-effect in final free recall for across-lists effects followed that introduced by Polyn *et al*.^6^ for measuring the effect in free recall. Using this measure, which we refer to as a *Temporal-clustering score*, we obtained a single value per participant and per condition (R, K) that reflects the utilization of temporal context. For each item recalled, all the absolute across-list lags of all possible transitions were calculated. Across-list lags refer to the distance between the lists in which two items were presented at study. We focused on across-list lags, regardless of the items’ within-list position. Transitions within the same list received a lag of zero. All of the possible lags were given a Spearman’s rank based on the absolute across-list lag (with the lowest lag given the highest rank). Following this transformation, each transition received a Temporal-clustering score between 0 and 1 based on the following equation: $\frac{R-1}{N-1}$, where R is the rank of the actual transition made and N is the number of possible transitions that could have been made. After scoring all of the transitions we average them into a single score of organization for each participant, separately for R and K. These scores have values between 0 and 1, with a score of 0.5 revealing that half of the times a participant made ‘context-utilized’ transitions and half of the time she did not, thus indicating chance-level organization which did not take into account the temporal context. A Temporal-clustering score of 1 indicates that all of the transitions received the highest possible score in terms of temporal organization.

#### Item-related-clustering-effect

This effect pertains to the judgment made during encoding, whether a word represented an abstract or a concrete entity. An Item-related-clustering Score was calculated as described in Polyn *et al*.^6^. We calculated for each list the proportion of transitions that were made between items with the same judgment (abstract or concrete) during encoding out of all the transitions made. To create a baseline measure, we used a relabeling technique in which we controlled for the proportion of judgments at both the list and participant level and randomly assigned labels to the items. We repeated the relabeling procedure 5,000 times and calculated the mean baseline Item-related-clustering score, taking into account the initial distribution of judgments made per participant and per list: for both R- and K-recalls, a difference score between the observed proportions and the baseline scores was calculated, this difference was the Item-related-clustering score. A positive score means that Item-related-clustering was utilized, whereas scores near zero imply that Item-related-clustering (abstract, concrete) was not utilized.

#### Semantic Clustering Effects

Semantic Clustering Scores were computed in a similar manner as that described for the Temporal-clustering score. Semantic distance was calculated using word2vec^58^ implemented with the ‘*Gensim’* software^59^ using a corpus of Hebrew Wikipedia articles supplied to us by the Hebrew Language Project^60^ at the National Institute for Testing and Evaluation (NITE; https://hlp.nite.org.il/). This corpus was collected on December 2012 and contains all the existing articles at that time (see Kennet and Anaki, 2017^61^ for further details).

#### Binning of transitions to R/K

For all clustering effects, the scores attributed to R/K refer to the score of the transition *from* an item judged as R/K. That is if item *i* was judged as R, the score of the transition between *i* to *i* + 1 (regardless of the judgment attributed to *i* + 1) was labeled as an R score. We then averaged all the R/K transition scores for each subject. This is consistent with our earlier definition of these transitions^17^.

The parametric statistical analyses of these effects included only subjects who had at least 3 valid recalls from each category (R/K). This was done in order to ensure that subject means were based on an adequate number of observations.

## Electronic supplementary material

Supplementary Information
