# Supplementary material for: A Remember/Know Examination of Free-recall Reveals Dissociative Roles of Item- and Context-Information over Time
Source: Sci Rep. 2018 Sep 10;8:13493. doi: 10.1038/s41598-018-31401-w (PMC6131345; doi:10.1038/s41598-018-31401-w)
Supplement: Supplementary file 1 — Supplementary Information [file 41598_2018_31401_MOESM1_ESM.docx]

Supplemental Information for:

**A Remember/Know Examination of Free-recall Reveals Dissociative Roles of Item- and Context-Information over Time**

Talya Sadeh, Rani Moran, Yonatan Stern and Yonatan Goshen-Gottstein

Temporal-context-effects: controlling for list-recency and fluctuations in attention

Two confounding factors— detailed by Howard et al. (2008)^1^—may influence the long-term Temporal-context-effect. First, it could be that an across-list Temporal-context-effect is affected by a large proportion of items that are retrieved from the most recent lists (i.e., the list-recency effect). In such a case, successive recalls would tend to be from nearby lists, all recalled with relatively high probability. Second, during the course of encoding, participants' attention may have fluctuated, such that for words in certain chunks of lists (e.g., lists 10-15) more attention and hence better encoding was given, as compared to words in other lists. Such fluctuations in attention would have increased the tendency of participants to successively recall words from nearby lists— lists constituting the chunks which received more attention—as compared to words from spaced-out lists. This would have, in turn, also affected the Temporal-context-effect.

To control for the possible effects of list-recency and attention, we used the approach described by Howard et al. (2008)^1^. This approach is based on the notion that if the across-list Temporal-context-effect is accounted for by list-recency or is a product of fluctuating attention, we would expect a similar magnitude of across-list Temporal-context-effects for a surrogate data set in which the output order of all recalls from the empirically-derived data was randomly shuffled—that is, we shuffled the order in which items were recalled in the final free-recall test. To illustrate, let us take an extreme situation, whereby recall is a product of list-recency, wherein attention was paid to only the last lists. In this situation, all transitions would be from the last two lists, yielding a strong across-list Temporal-context-effect for lag +1 and lag -1. Under this hypothetical scenario, shuffling the output order would yield surrogate data sets in which only transitions across the last two lists would be found, again producing a strong across-list Temporal-context-effect for lag +1 and lag -1. Hence, to the extent that our data are compatible with the surrogate data, it cannot be ruled out that they only reflect a product of attention and list-recency rather than a pattern of true retrieval dynamics. If, however, our data reflect a true pattern of retrieval dynamics, whereby recall of a word in final free-recall tends to follow recall of a word from a nearby list—rather than list-recency or attention—the Temporal-context-effect in the empirical data would be significantly stronger than the Temporal-context-effects of such a surrogate data set.

To create the surrogate data set, we randomly-shuffled the output order of all recalls for each participant, while maintaining their original R/K labeling and their number of list in which they were studied. Temporal-context-effects were then computed for R and K-recalls. An 'R recall' was defined as a transition *from* an R item to any item (either K or R) and a 'K-recall', as one from a K item to any item. This is consistent with our definition of these transitions in the Main Text. Finally, the across-participant mean difference between R and K Temporal-clustering scores was calculated.

We repeated the procedure described in the paragraph above 5,000 times. This resulted in a distribution of 5,000 mean R-K difference scores, under the null hypothesis that the difference between R and Ks Temporal-clustering-effects is merely a product of attention and list-recency. The difference between the Temporal-clustering of R and K in the original, empirically-derived dataset was 0.072 and was larger than 4,946 (98.9%) out of the 5,000 surrogate-data scores. Thus, the empirically-derived results deviate from the distribution under the null hypothesis, with a significance level of p = .011.

**REFERENCES**

1 Howard, M., Youker, T. & Venkatadass, V. The persistence of memory: Contiguity effects across hundreds of seconds. *Psychonomic Bulletin & Review* **15**, 58-63 (2008).
